# Lipidomics of human umbilical cord serum: identification of unique sterol sulfates

**DOI:** 10.4155/fsoa-2017-0012

**Published:** 2017-04-05

**Authors:** Paul L Wood, Heli Siljander, Mikael Knip

**Affiliations:** 1 Metabolomics Unit, Department of Physiology & Pharmacology, DeBusk College of Osteopathic Medicine, TN, USA; 2 College of Veterinary Medicine, Lincoln Memorial University, 6965 Cumberland Gap Pkwy., Harrogate, TN 37752, USA; 3 Children’s Hospital, University of Helsinki & Helsinki University Hospital, Helsinki, Finland; 4 Research Programs Unit, Diabetes & Obesity, University of Helsinki, Helsinki, Finland; 5 Folkhälsan Research Center, University of Helsinki, Helsinki, Finland; 6 Department of Pediatrics, Tampere University Hospital, Tampere, Finland

**Keywords:** bile acid sulfates, glycerophospholipids, lipidomics, neurosteroid sulfates, perfluoroalkyl toxins, umbilical cord serum

## Abstract

**Aim:**

There are currently limited lipidomics data for human umbilical cord blood. Therefore, the lipidomes of cord sera from six newborns and sera from six nonpregnant females were compared.

**Materials & methods:**

Sera lipidomics analyses were conducted using a high-resolution mass spectrometry analytical platform.

**Results:**

Cord serum contained a diverse array of glycerophospholipids, albeit generally at lower concentrations than monitored in adult serum. The unexpected observations were that cord serum contained several neurosteroid sulfates and bile acid sulfates that were not detectable in adult serum.

**Conclusion:**

Our data are the first to demonstrate that cord serum contains bile acid sulfates that are synthesized early in the hydroxylase, neutral and acidic pathways of primary bile acid biosynthesis and support previous publications of cord blood perfluoralkyl toxins in newborns.

## Lay abstract

Umbilical cord blood offers the potential to increase our understanding of fetal development during pregnancy and during development after delivery. Our studies of complex sterols in umbilical cord blood (bile acid sulfates) suggest that with further studies these may be useful biomarkers of abnormal fetal liver development.

The vessels of the umbilical cord supply the fetus with oxygen and nutrients, and remove fetal waste products. Even though percutaneous umbilical cord blood sampling has been an established method for obtaining samples for fetal genetic testing during the last decades, limited evaluation of umbilical cord blood lipidomics has been undertaken thus far [1,2]. To define the lipidomics differences between the lipid profiles of cord sera and adult female sera, we undertook a nontargeted pilot study of cord lipidomics utilizing a high-resolution mass spectrometric platform. This involved an evaluation of cord sera from six healthy newborns and compared their lipid profiles with those of the healthy nonpregnant adult females. Nonpregnant adult females were chosen as the controls so that the baseline measurements were not complicated by the fluid communications between the fetus and a pregnant mother. Our data add to the human cord blood lipidomics database covering glycerophospholipids, sphingolipids, neutral lipids and free fatty acids.

Cord serum contained a diverse array of glycerophospholipids, albeit generally at lower concentrations than monitored in adult serum. No major differences in the diversity or levels of sphingolipids were detected. In contrast, cord serum levels of neutral lipids (diacylglycerols [DAG] and triacylglycerols [TAG]) were dramatically lower than adult serum. Levels of free fatty acids were similar. We were also able to describe for the first time that cord serum contains levels of bile acid sulfates not detected in adult serum.

## Materials & methods

### Blood collection & processing

Umbilical cord arterial blood was collected at parturition, according to an Ethics Committee-approved protocol. Serum was collected and frozen at -80°C prior to sample processing. Blood was also collected for three male and three female newborn infants. Control adult female serum was collected from six nonpregnant women (age 32–40 years).

### Lipidomics analyses of serum

Lipid profiles were analyzed for all serum samples as follows. After thawing, 100 μl aliquots of serum were mixed with 1 ml of methanol containing stable-isotope internal standards [3,4]. Next, 1 ml of water and 2 ml of methyl-tert-butyl ether were added and the tubes were vigorously shaken at room temperature for 30 min. After centrifugation at 3000 × g for 10 min, 1 ml of the upper organic layer was dried by centrifugal vacuum evaporation prior to dissolution in 150 μl of isopropanol:methanol:chloroform (4:2:1) containing 15 mM ammonium acetate. Shotgun ESI lipidomics (5 μl/min) was performed utilizing high-resolution data acquisition (140,000 at 200 amu; 0.3–3 ppm mass error; *m/z* 200–1400) with an orbitrap mass spectrometer (Thermo Q Exactive).

In negative-ion ESI (3.2 kV, capillary temperature of 320°C, sheath gas of 10), the anions of ethanolamine plasmalogens (PlsEtn), phosphatidylglycerols (PG), phosphatidylinositols (PtdIn), sterol sulfates, free fatty acids and perfluoroalkyl toxins, and the [M + HCOO]^-^ anions of ceramides were monitored. In positive-ion ESI (4.3 kV, capillary temperature of 320°C, sheath gas of 10), the cations of choline plasmalogens (PlsCh), phosphatidylcholines, ceramides and sphingomyelins (SM) and the ammonium adducts of DAG and TAG were monitored.

Data are presented as R values (ratio of the endogenous lipid peak area to the peak area of an appropriate internal standard) per 100 μl of cord or adult serum (n = 6).

### Identification of unknowns

The exact masses of unknown metabolites were used to interrogate public databases for compound identification. These databases included LipidMaps, hmdb and Kegg. Putative metabolites were subsequently validated by MS^2^. Putative aldehyde metabolites were derivatized with Girard’s reagent T (GRT). To the dried methyl-tert-butyl ether extracts, added 0.1 ml of reagent (20 mg GRT per ml of methanol) and 20 μl of acetic acid prior to heating at 70°C for 30 min with shaking. The samples were dried and dissolved in infusion solvent. Aldehydes were augmented in mass by 113.09528 after derivatization with GRT and the [M+H]^+^ ions of these derivatives were monitored in ESI.

### Statistics

Data were analyzed utilizing the two-tailed t-test assuming unequal variances (Excel).

## Results

### Gender differences

There were no gender differences in the umbilical cord plasma levels of lipids or sterol sulfates between newborn males and females. However, this study had only three samples for each gender.

### (O-acyl) ω-hydroxy-fatty acids

(O-acyl) ω-hydroxy-fatty acids, previously detected in amniotic fluid [3], were not detected in human cord blood.

### Choline glycerophospholipids

**Figure 1.**
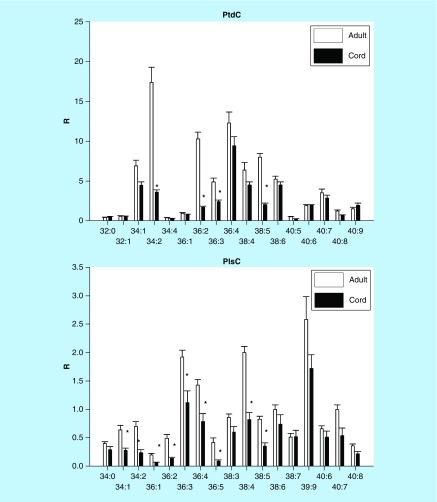
**Umbilical cord serum levels of phosphatidylcholines and choline plasmalogens.** Mean ± SD. The nomenclature refers to the fatty acid or fatty alcohol substitutions at sn-1 and sn-2 of the glycerol backbone. For example, 32:1 refers to 32 carbons with one double bond. *p < 0.01. PlsC: Choline plasmalogen; PtdC: Phosphatidylcholine; R: Ratio of peak area of endogenous lipid to the peak area of the internal standard.

Cord serum was observed to possess a similar diversity of diacylglycerophosphocholines (phosphatidylcholines) and PlsCh as monitored in adult serum (Figure 1). A number of choline glycerophospholipids were monitored at 50–80% lower levels in cord serum, except for lipids with polyunsaturated fatty acid substitutions with six to nine unsaturated bonds.

### Ethanolamine glycerophospholipids

**Figure 2.**
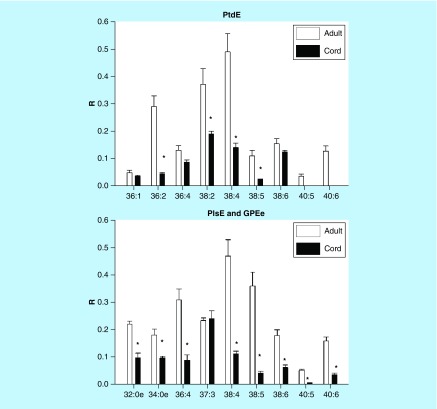
**Umbilical cord serum levels of phosphatidylethanolamines, ethanolamine plasmalogens and 1-alkyl-2-acyl glycerophosphoethanolamines.** Mean ± SD. The nomenclature refers to the fatty acid or fatty alcohol substitutions at sn-1 and sn-2 of the glycerol backbone. For example, 36:4 refers to 36 carbons with four double bonds. *p< 0.01 GPEe: 1-alkyl-2-acyl glycerophosphoethanolamine; PlsE: Ethanolamine plasmalogen; PtdE: Phosphatidylethanolamine; R: Ratio of peak area of endogenous lipid to the peak area of the internal standard.

Phosphatidylethanolamines, alkyl-acyl-phosphatidylethanolamines and PlsE were 50–70% lower in cord serum than in adult serum (Figure 2).

### Phosphatidylinositols & phosphatidylglycerols

**Figure 3.**
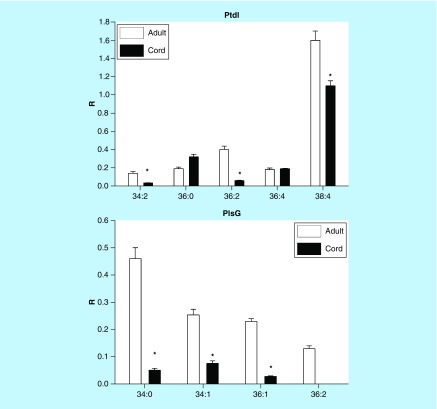
**Umbilical cord serum levels of phosphatidylinositols and phosphatidylglycerols.** Mean ± SD. The nomenclature refers to the fatty acid substitutions at sn-1 and sn-2 of the glycerol backbone. For example, 34:3 refers to 34 carbons with three double bonds. *p < 0.01. PtdG: Phosphatidylglycerol; PtdI: Phosphatidylinositol; R: Ratio of peak area of endogenous lipid to the peak area of the internal standard.

PtdIn levels were generally lower in cord serum while PG levels were 50–60% less than measured in adult serum (Figure 3).

### Neutral lipids

**Figure 4.**
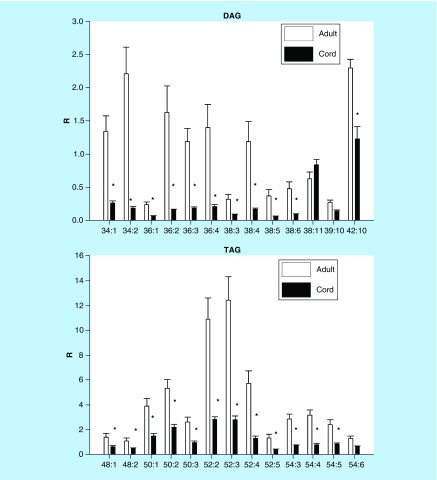
**Umbilical cord serum levels of diacylglycerols and triacylglycerols.** Mean ± SD. The nomenclature refers to the fatty acid substitutions of the glycerol backbone. For example, 34:1 refers to 34 carbons with one double bond. *p < 0.01. DAG: Diacylglycerol; R: Ratio of peak area of endogenous lipid to the peak area of the internal standard; TAG: Triacylglycerol.

DAG and TAG levels were 50–80% lower in cord serum (Figure 4).

### Sphingolipids

**Figure 5.**
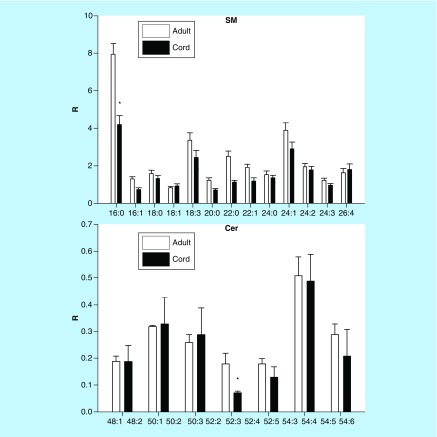
**Umbilical cord serum levels of sphingolipids.** Mean ± SD. The nomenclature refers to the fatty acid substitution of the sphingosine backbone. For example, 16:0 refers to palmitic acid. *p < 0.01. Cer: Ceramide; R: Ratio of peak area of endogenous lipid to the peak area of the internal standard; SM: Sphingomyelin.

SM and ceramide levels were similar for cord and adult serum with few exceptions showing lower levels in cord blood (Figure 5).

### Free fatty acids

**Figure 6.**
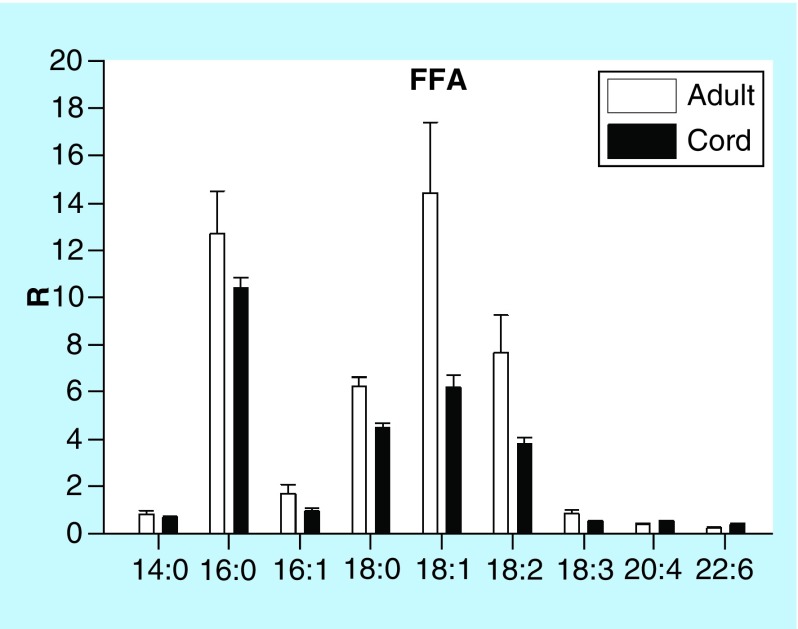
**Umbilical cord serum levels of free fatty acids.** Mean ± SD. *p < 0.01. FFA: Free fatty acid; R: Ratio of peak area of endogenous lipid to the peak area of the internal standard.

Free fatty acid levels of cord serum were similar to those monitored in adult serum (Figure 6).

### Sterol sulfates

Cord serum was found to contain a diverse array of neurosteroid sulfates not detected in adult serum (Figure 6). These included pregnenolone sulfate, hydroxypregnenolone sulfate, allopregnanolone sulfate and tetrahydrodeoxycorticosterone sulfate. Dehydroepiandrosterone sulfate was detected in both cord and adult plasma, but shotgun lipidomics cannot distinguish dehydroepiandrosterone sulfate from testosterone sulfate.

The bile acids, taurodeoxycholic acid and glycochenodeoxycholic acid sulfate were found at lower levels in cord serum than in adult serum. However, several sulfates of bile acid intermediates were only detected in cord serum. MS^2^ of the sterol sulfates all yielded a strong product anion of 96.9595 [HSO_4_]^-^ with 0.10–0.21 ppm mass error, supporting that these biomarkers were sulfates. The monitored [M-H]^-^ anion of 497.2942 could be dihydroxycholesterol sulfate, cholestenetriol sulfate (24 and 25 triol) and/or dihydroxycholestanal sulfate. Derivatization with GRT did not alter the mass of 497.2942, eliminating the possibility that this sterol sulfate was the aldehyde. Therefore, the mass 497.2942 is most likely dihydroxycholesterol sulfate in the acidic pathway and/or cholestenetriol sulfates (24/25 triols) in the hydroxylase pathways of bile acid biosynthesis.

Similarly, the [M-H]^-^ anion of 513.2892 could be dihydroxycholestanoate sulfate and/or trihydroxycholestanal sulfate. Derivatization with GRT did not alter the mass of 513.2892, eliminating the possibility that this sterol sulfate was the aldehyde. Therefore, the mass 513.2892 is most likely dihydroxycholestanoate sulfate in the neutral pathway of bile acid biosynthesis.

### Perfluoroalkyl environmental/industrial toxins

The industrial toxins and perfluoro-octane sulfonate (PFOS), perfluorononanoic acid, perfluoro-octanoic acid and perfluorohexane sulfonic acid were found in adult serum, while only PFOS and perfluorononanoic acid were detected in cord serum, albeit at much lower concentrations in the case of PFOS (Table 1). MS^2^ of PFOS and perfluorohexane sulfonic acid yielded a strong product anion of 79.9568 [SO_3_]^-^ with 2.12 ppm mass error, supporting that these were sulfonic acids.

**Table 1.** **Sterol sulfates and environmental perfluoroalkyl toxins monitored in adult and umbilical cord serum.**

**Sterol sulfate or perfluoroalkyl toxin**	**Calculated [M-H]^-^**	**Observed ppm**	**Adult serum**	**Cord serum**
			**Mean ± SD (range)**	**Mean ± SD (range)**
Cholesterol sulfate	465.3044	0.68	0.94 ± 0.13	1.11 ± 0.29
DHEAS/testosterone sulfate	367.1585	0.94	0.86 ± 0.36	1.40 ± 0.13^†^
Pregnenolone sulfate	395.1898	0.39	ND	0.89 ± 0.18
Hydroxypregnenolone sulfate	411.1847	1.48	ND	0.86 ± 0.26
Allopregnanolone sulfate	397.2054	1.75	ND	0.84 ± 0.035
THDOC sulfate	413.2003	1.92	ND	0.67 ± 0.093
Glycochenodeoxycholic acid sulfate	528.2637	1.82	0.43 ± 0.12	0.18 ± 0.057^†^
Taurodeoxycholic acid	498.2895	1.19	0.14 ± 0.016	0.085 ± 0.019^†^
Cholestenetriol/dihydroxycholesterol sulfate	497.2942	1.19	ND	0.42 ± 0.096
Dihydroxycholestanoate sulfate	513.2892	1.05	ND	0.20 ± 0.029
Perfluoro-octane sulfonic acid	498.9302	1.23	2.30 ± 1.9 (0.55–5.0)	0.39 ± 0.08^†^ (0.30 – 0.50)
Perfluorononanoic acid	462.9632	1.91	0.44 ± 0.19 (0.31–0.71)	0.24 ± 0.10 (0.11–0.36)
Perfluorooctanoic acid	412.9664	0.93	0.36 ± 0.35 (0.04–0.96)	ND
Perfluorohexane sulfonic acid	398.9366	0.97	0.41 ± 0.34 (0.11–0.98)	ND

Values (mean ± SD) represent the ratio of the peak area of the endogenous sterol sulfate/peak area of 100 pmol of the internal standard [^2^H_7_]cholesterol sulfate per 0.1 ml of cord serum.

^†^p < 0.05 vs adult plasma.

DHEAS: Dehydroepiandrosterone sulfate; ND: Not detected; ppm: Parts per million mass error; THDOC: Tetrahydrodeoxycorticosterone.

## Discussion

As limited analyses of the umbilical cord serum lipidome have previously been available [1,2], we undertook a detailed lipidomics analysis of cord serum. Unsurprisingly, we found that the umbilical cord serum lipidome is dominated by similar lipids to those circulating in the maternal serum. Our data indicate that in cord sera, the diversities of inositol, choline and ethanolamine glycerophospholipids are comparable to the diversities observed in adult sera, but that a number of these lipids are present at lower levels in the cord blood. Our data are the first to demonstrate the presence of 1-alkyl-2-acyl glycerophosphoethanolamines (GPE 32:0e and GPE 34:0e) in cord sera. The lower levels of 1-alkyl-2-acyl glycerophosphoethanolamines, PlsE and PlsC in cord blood strongly suggest that peroxisomal function, which is responsible for the biosynthesis of ether lipids [5], is active in the fetus [6] and that these vinyl-ether lipids are not exported into the cord blood but are utilized as essential membrane lipids by the fetus.

Cord sera also possessed significantly lower circulating levels of PtdG and PtdI than are present in adult sera. Similarly the neutral lipids (DAG and TAG) were much lower in cord sera. This may indicate that the fetal liver biosynthesis of these lipids is sufficient for fetal metabolism but not for export to the cord blood. Our data also suggest that the fetal liver is the main source of DAG and TAG essential for energy metabolism, signal transduction and biosynthesis of glycerolipids and glycerophospholipids [4].

The levels of free fatty acids and the sphingolipid lipidome profile (levels of SM and ceramides) of the cord sera were comparable to the ones observed in adult sera. These data possibly imply that free fatty acids and sphingolipids may pass freely between maternal and fetal circulations.

Bile acids of the cord sera are generally considered to derive from the fetus rather than the mother [7,8]. Polyhydroxylated bile acids and bile acid sulfates are not present in nonpregnant healthy adults, but appear in the serum of patients with inborn errors of bile acid metabolism [8–10] or with cholestatic liver diseases [7]. During pregnancy, polyhydroxylated bile acids produced by the fetal liver are transported via cord blood to the mother for further metabolism and elimination [7,8]. Concentrations of these polyhydroxylated bile acids decrease in healthy infants between the age of 7 years and 30 days *postpartum* [11]. Our data demonstrate that sulfates of some of these polyhydroxylated bile acids are excreted into the cord blood, presumably via the cations of cytosolic sulfotransferases which catalyze sulfation of hydroxylated sterols [9]. Specifically, sterol sulfates, including cholestenetriol and dihydroxycholestanoate sulfates, were not detected in the sera of the nonpregnant adult females. Elevated levels of these bile acid sulfates have been reported in infants with hydroxysteroid dehydrogenase deficiency [9–12] indicating that these may be useful biomarkers for abnormal fetal liver development.

Even though cord neurosteroids have been suggested to originate from the mother rather than the fetus [13–17], we observed that in the cord blood there were a number of neurosteroid sulfates which were absent in the adult sera. Supply of neurosteroids and neurosteroid sulfates is critical for the neurological development of the fetus, since they are potent modulators of GABAergic neuronal transmission [18,19] and are involved in synaptic plasticity and neuronal development [19–21]. It remains to be determined if the neurosteroid sulfates we monitored originate from the fetus or from the mother.

We confirmed previous findings that perfluoroalkyl-containing environmental toxins are present both in cord sera and in adult sera [22–26]. Analyzing cord blood for perfluoroalkyl toxins may be a useful means of determining fetal exposure to these carcinogenic environmental toxins, which have a detrimental effect on birthweight and sexual development of the fetus and young infant. The major limitation of this study is the small sample size. In addition, chromatographic methods should be utilized next to validate the identification of the sterols and perfluoroalkyl compounds.

## Conclusion

In summary, our pilot study significantly expands our knowledge database for the lipidome of human cord sera and suggests that several potential biomarkers are worthy of further study. These include bile acid sulfates as potential biomarkers of abnormal fetal liver function, neurosteroid sulfates as potential biomarkers of fetal neural development and perfluoroalkyl-containing environmental toxins as potential biomarkers of fetal risks for slow development and early cancer development.

## Future perspective

These data suggest that future studies of bile acid sulfates during *in utero* and *postpartum* development may lead to new diagnostics for evaluating normal versus abnormal liver development. Inclusion of pregnant and nonpregnant females also will be valuable comparisons in future studies.

**Executive summary**

- Sterol sulfates, including cholestenetriol and dihydroxycholestanoate sulfates, were detected in umbilical cord blood but not in the sera of the nonpregnant adult females, suggesting that bile acid metabolism in the fetus is more complex than in adults.
- Perfluoroalkyl toxins in umbilical cord blood may provide useful indices of the exposure of the fetus to these dangerous toxins.
